# Quantification of Recruit Training Demands and Subjective Wellbeing during Basic Military Training

**DOI:** 10.3390/ijerph19127360

**Published:** 2022-06-15

**Authors:** Sean Bulmer, Jace R. Drain, Jamie L. Tait, Sean L. Corrigan, Paul B. Gastin, Brad Aisbett, Timo Rantalainen, Luana C. Main

**Affiliations:** 1 Centre for Sport Research, School of Exercise and Nutrition Sciences, Deakin University, Geelong 3220, Australia; bulmerse@deakin.edu.au (S.B.); s.corrigan@deakin.edu.au (S.L.C.); 2 Defence Science and Technology Group, Fishermans Bend 3207, Australia; jace.drain@defence.gov.au; 3 Institute for Physical Activity and Nutrition (IPAN), School of Exercise and Nutrition Sciences, Deakin University, Geelong 3220, Australia; j.tait@deakin.edu.au (J.L.T.); brad.aisbett@deakin.edu.au (B.A.); 4 La Trobe Sport and Exercise Medicine Research Centre, School of Allied Health, Human Services and Sport, La Trobe University, Bundoora 3083, Australia; p.gastin@latrobe.edu.au; 5 Gerontology Research Centre and Faculty of Sport and Health Sciences, University of Jyväskylä, 40014 Jyväskylä, Finland; timo.rantalainen@jyu.fi

**Keywords:** soldier, army, self-report, monitoring, recruit, allostatic load

## Abstract

Purpose: Assess and describe the physical demands and changes in subjective wellbeing of recruits completing the 12 week Australian Army Basic Military Training (BMT) course. Methods: Thirty-five recruits (24.8 ± 6.8 y; 177.4 ± 10.1 cm, 75.6 ± 14.7 kg) consented to daily activity monitoring and weekly measures of subjective wellbeing (Multi-component Training Distress Scale, MTDS). The physical demands of training were assessed via wrist worn activity monitors (Actigraph GT9X accelerometer). Physical fitness changes were assessed by push-ups, sit-ups and multi-stage shuttle run in weeks 2 and 8. Results: All objective and subjective measures significantly changed (*p* < 0.05) across the 12 week BMT course. In parallel, there was a significant improvement in measures of physical fitness from weeks 2 to 8 (*p* < 0.001). The greatest disturbance to subjective wellbeing occurred during week 10, which was a period of field training. Weeks 6 and 12 provided opportunities for recovery as reflected by improved wellbeing. Conclusions: The physical demands of training varied across the Australian Army 12 week BMT course and reflected the intended periodization of workload and recovery. Physical fitness improved from week 2 to 8, indicating a positive training response to BMT. Consistent with findings in sport, wellbeing measures were sensitive to fluctuations in training stress and appear to have utility for individual management of personnel in the military training environment.

## 1. Introduction

Basic Military Training (BMT) provides recruits with foundational training, preparing them for the rigors of military service. During BMT, recruits are exposed to a variety of physical and cognitive demands, including a newly regimented lifestyle, new social and environmental stressors, and the constant pressure of instructor and peer assessment [1,2,3]. Recruits must meet a range of military competencies and standards to graduate from BMT and progress to trade-specific training (e.g., infantry, logistics, armored). The development of military-relevant physical capacity is therefore fundamental to many of these training outcomes. However, high physical training volumes, insufficient recovery opportunities, and increased stress during military training have been linked to a number of maladaptive outcomes, such as musculoskeletal injury, illness and training attrition [4]. These negative outcomes increase training costs, burden the healthcare system, increase future injury risk, and can impair career progression [5]. Maladaptation to a training stimulus can be caused by combinations of a sudden increase in training load, high total training load, inadequate recovery, training monotony or environmental and lifestyle stressors [6]. Avoiding maladaptation and achieving time-efficient physical performance gains with the lowest possible injury rate is therefore essential for a successful, sustainable military [7].

Traditionally, military physical training programs have favored cardiorespiratory and muscular endurance training [7,8]. Steady-state running, long-distance marching, and circuit training have made up a substantial portion of the prescribed workload [2,8,9]. In comparison, the development of muscular strength, and the provision of deliberate recovery periods have been a lesser focus [8,9]. This combination of moderate to high volume training and inadequate recovery has been associated with symptoms of maladaptation and increased injury incidence [7]. Contemporary military training recommendations have subsequently specified that physical training (PT) programs include focused strength training [9] within a periodised program that provides progressive overload coupled with adequate recovery [7]. In 2015, the Australian Army adopted a more contemporary PT program that incorporated muscular strength and high-intensity interval training, to better align with occupational demands and mitigate the risk of preventable musculoskeletal injuries. For practicality, this PT program is implemented in a group training format, with some opportunity for individualization to help ensure equivalent relative intensity across the group. However, it is unlikely that the group-delivered training stimuli will be optimal for all recruits, given variances in fitness and training history [10]. Moreover, group-level training prescription without strategies to monitor individual recruits’ training strain could lead to sub-optimal training adaptations, unsustainable loads or adverse events (i.e., injury).

While PT is an obvious source of training demands during BMT, from a scheduling perspective, PT lessons represent <10% of the total lesson time in the Australian Army BMT program [9], which is similar to other BMT courses [2]. In addition to PT, there are various military skill and education lessons (e.g., patrolling, offensive and defensive ground maneuvers, navigation, drill, marching) that similarly impose physical and physiological demands. On top of these physical demands are psychological and cognitive challenges, and disrupted sleep, all collectively contributing to high allostatic loads [1,11,12]. Consequently, given previously observed high rates of musculoskeletal injury and training attrition, there would be measurable benefits associated with the implementation of an ongoing monitoring system for the management of the training demands imposed on recruits undertaking BMT.

Optimization of high loads in a group training environment is a challenge that BMT shares with elite team sports. In sport organizations, consistent and actionable information for load management is routinely gathered via self-report monitoring strategies that track changes in athlete wellbeing [13]. These leverage an individual’s perception of their wellbeing over time, to identify athletes at risk of poor training outcomes or compromised performance. Subsequent training modifications and interventions prescribed based on known relationships between training load and stress responses can mitigate the risk of these negative outcomes [14]. Self-report measures of subjective wellbeing are widely used for their cost-effectiveness and for the minimal time required to administer and analyze the results, which makes them practical for large organizations to implement. These measures characterize an individual’s subjective state in distinct domains such as stress, depressed mood states and physical ailments (e.g., muscle and joint soreness/pain) relating to physical and mental wellbeing. Scales measuring changes in multiple domains at once (i.e., mood, perceived stress, symptom checklists and recovery status) provide a more holistic picture of an individual’s perception of their wellbeing, when compared with measures from a single domain [15]. Such measures are gaining traction, as they provide immediate insights into the most affected domains and identify areas that should be prioritized for intervention. To date, the demands of training during the recently modified Australian Army BMT program and the program’s effects on wellbeing have not been quantified. Therefore, the aims of the current study were to (1a) assess and describe the physical demands imposed on recruits during BMT; (1b) quantify subjective wellbeing of recruits during BMT; and (2) explore relationships between the physical demands and subjective wellbeing during BMT.

## 2. Methods

### 2.1. Participants

Participants were Australian Army recruits commencing the 12-week BMT program at the Army Recruit Training Centre, Blamey Barracks, Kapooka, Australia in July 2018. The recruits were from two platoons within the same training company and commenced BMT on the same day. Potential participants were verbally briefed and provided a written project description by an investigator in accordance with the protocol approved by the Department of Defence and Veterans’ Affairs Human Research Ethics Committee (protocol number: 021-17). No uniformed personnel were present for the briefing, and it was made clear that participation was voluntary and would have no bearing on current or future career opportunities. Written informed consent to participate was provided by 48 recruits, and descriptive data is provided in Table 1. All participants met the Australian Army enlistment fitness requirements of 45 sit-ups with feet held, a 7.5 multi-stage shuttle run score, and 8 (female) or 15 (male) push-ups.

### 2.2. Experimental Design and Procedures

Following the provision of consent during the first week of BMT, a baseline physical activity questionnaire, the International Physical Activity Questionnaire (IPAQ) Short Form was completed within 48 h. The questionnaire consists of four questions (with embedded sub-questions) relating to vigorous, moderate, and walking physical activity, as well as sedentary behavior performed in the past seven days [16]. The IPAQ results showed that per day prior to BMT, recruits spent a daily average of 4.8 h sitting, 1.7 h walking, 1 h at moderate intensities, and 0.8 h at vigorous intensities. Anthropometric data was gathered from the Army’s initial screening of the recruits by health center nursing staff.

For the duration of BMT, participants wore an ActiGraph GT9x (ActiGraph, Pensacola, FL, USA) on their non-dominant wrist. Units were set at a collection rate of 30 Hz using 60 s epochs. Daily steps and physical activity counts were used to quantify the physical demands of training. Activity counts were converted to a daily estimation of energy expenditure (EE) via the method of neural network, including absolute acceleration values and ‘feature set 1′ for wrist-independent EE estimation, as described by Montoye et al. [17]. The calculated METs per day for each participant were summed to report daily MET minutes, with the weekly total energy expenditure being the accumulation of MET minutes from Monday to Sunday each week.

These results are reported as weekly energy expenditure (MET minutes) and % weekly total time in zones for sedentary, light, moderate, vigorous, as well as moderate and vigorous physical activity (MVPA) [18]. A minimum of five days of data per week per participant was required for inclusion in the physical activity analysis per week. Due to having less than a full week of data after consent was granted, week 1 accelerometer data were not included in the analysis. Participants wore the ActiGraphs for the entire duration of the study, except for any periods of water immersion (i.e., showering or swimming), and to charge the units. The devices were collected by research staff Monday night prior to bedtime; the data were downloaded, and the devices charged, prior to their return to participants upon waking Tuesday morning. Non-wear time was determined as more than three hours of consecutive ‘0′ total acceleration per day, except Monday and Tuesday, where eight hours was used to account for downloading and charging time. The 3 h non-wear threshold is more stringent than the standard minimum 10 h/day total wear time often used in free-living adults [19].

Subjective assessment of training demands was quantified via Rating of Perceived Exertion (RPE) [20] prior to bed each day as close to 10:00 p.m. as practicable using pen and paper. RPE is an indicator of exertion and was used to add context to EE each day. Participants used a category ratio scale (CR-10) of 0 to 10 to rate their exertion for the day where 0 = ‘nothing at all’, 2 = ‘weak’, 5 = ‘strong’, 7 = ‘very strong’ and 10 = ‘very, very strong’. While this measure has historically been used to rate a single training ‘session’, it has been previously utilized as a daily measure in military settings [21,22].

Subjective wellbeing was assessed using the Multi-component Training Distress Scale (MTDS), which is a validated measure for athlete populations [15] and has previously been used for firefighters [23]. The short 22-item measure was completed via pen and paper upon wakening every Sunday morning for the duration of the study. Twenty-two items, grouped under six factors: depression, vigor, physical symptoms, sleep disturbances, stress and fatigue were answered on a five-point scale (zero to four: 0 = ‘not at all’, 1 = ‘a little’, 2 = ‘moderately’, 3 = ‘quite a bit’, 4 = ‘extremely’). Internal consistency of the subscales have previously been reported with Cronbach alpha ranging from 0.72 to 0.86 [15].

Physical fitness was assessed in weeks 2 and 8 of BMT. The physical fitness tests included maximum push-ups and sit-ups (feet held) in two minutes, and the multi-stage shuttle run (i.e., the Beep test) [9]. Multi-stage shuttle run performance was used to predict VO_2peak_ [24].

### 2.3. Data Analysis

All statistical analyses were performed using SPSS software (IBM SPSS Statistics for Windows, Version 26.0, 2019, Armonk, NY, USA). Participants with data from ≥75% of BMT were included in the analysis to adequately represent BMT. All data and their residuals were checked for normality. To determine changes in physical fitness, a repeated measure ANOVA was conducted. In cases where Mauchly’s tests of sphericity indicated that sphericity had been violated (*p* < 0.001), multivariate tests are reported in the form of the Huynh–Feldt correction. Where there was a significant change over time, post hoc analyses were conducted using the Bonferroni adjustment. For assessing the changes in subjective wellbeing and training demands across the twelve weeks of BMT (aim 1a and 1b), linear mixed models were used. Pairwise comparisons with a significance level of *p* = 0.05 using a Bonferroni correction were utilized if a main effect of time was present (*p* < 0.05).

To explore relationships between training demands and subjective wellbeing (Aim 2), linear mixed models were employed. For subjective wellbeing, the MTDS total score was utilized as the outcome variable. A first order autoregressive covariance structure was utilized for the repeated measures of timepoints, due to a reduction in covariance for increasing timepoint lags of the unstructured matrix. Both singular predictors and an overall model of best fit were used to determine which indices of training demands and individual characteristics predicted MTDS total score. For singular predictors, each variable in solidarity was assessed on its predictive capacity of MTDS total score. For the model of best fit, all possible models comprising combinations of indices of training demands, physical fitness, age, gender, and BMI variables were created. Models were compared to achieve the lowest Akaike’s Information Criterion (AIC), an indication of model fit that accounts for model parsimoniousness. The fixed effects of the most parsimonious model with the lowest AIC are reported. For fixed effects of the individual predictors and the overall model of best fit, F-statistics (F), coefficients (ß) and *p* values (p) are reported with the significance set a *p* = 0.05.

## 3. Results

**Figure 1:**
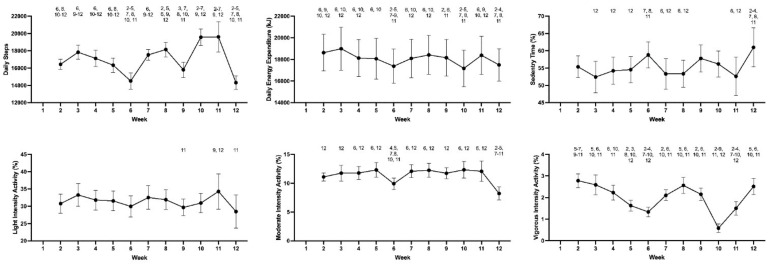
Physical activity measures across the 12 week BMT program (mean ± 95% confidence intervals). Note: Numbers above data points denote a significant difference to the detailed week (*p* < 0.05).

**Figure 2:**
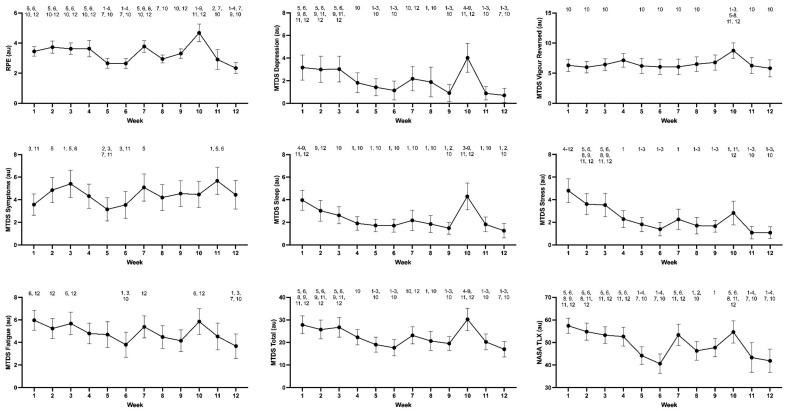
Changes in subjective assessments of training demands and wellbeing across the 12-week BMT program (mean ± 95% confidence intervals). Note: RPE, rating of perceived exertion; MTDS, multi-component training distress scale; NASA TLX, National Aeronautical Space Agency task load index; au, arbitrary units. Numbers above data points denote a significant difference to the detailed week (*p* < 0.05).

Of the 48 recruits who participated in the study, 35 recruits (29 men and 6 women; aged 24.8 ± 6.8 y; height 177.38 ± 10.1 cm; body mass 75.6 ± 14.7 kg) had data for ≥75% of BMT and were included in the analysis. All markers of training demands changed (*p* < 0.05) during BMT. Pairwise comparisons between weeks for physical activity, subjective assessments of training demands and wellbeing are depicted in Figure 1 and Figure 2. Physical fitness as measured by push-ups, sit-ups, and predicted VO_2peak_ improved between weeks two and eight. Participants completed 5.4 ± 1.7 more (*p* = 0.003) push-ups. Similarly, recruit sit-up performance also significantly improved (*p* < 0.001)*,* with participants average sit-up performance more than doubling during the same period of time. Estimated VO_2peak_, as measured by the beep test, also significantly increased over time (*p* < 0.001), with a mean improvement of 4.96 mL.kg^−1^.min^−1^.

Significant singular predictors of MTDS total included daily steps (F = 14.92, ß = 0.001, *p* < 0.001), % vigorous physical activity (F = 8.63, ß = −141.3, *p* = 0.004), NASA TLX (F = 46.08, ß = 0.26, *p* < 0.001), RPE (F = 33.65, ß = 2.03, *p* < 0.001) and gender (F = 6.01, ß = 9.54, *p* = 0.020); with females more likely to report higher MTDS total scores. The overall model of best fit for predicting MTDS total score is detailed in Table 2.

## 4. Discussion

This study is the first to systematically characterize the physical demands of training during the Australian Army BMT program (weeks 2–12), providing both accelerometry-derived and subjective measures. Accordingly, the primary aim of this study was to quantify the physical demands imposed on recruits, and changes in recruit responses (subjective wellbeing and physical fitness) across the 12 weeks of BMT. Results showed that recruits were exposed to high volumes of physical activity during BMT (~17,000 steps·day^−1^ and ~18 MJ·day^−1^). Results also indicated that all indices of the training demands changed significantly over the 12 weeks. Importantly, subjective wellbeing improved during BMT, with weeks 6 and 12 providing recovery opportunities. The second aim of this study was to explore relationships between the training demands and subjective wellbeing. Training demands were associated with subjective measures of wellbeing, supporting the potential utility of measures of subjective wellbeing to manage recruit strain during BMT, which is consistent with theoretical expectations.

Recruits averaged 17,031 steps per day during BMT, which is higher than recent studies in Australian, British, and U.S. recruits (10,000–14,000 steps per day) [9,21,25,26]. However, the physical activity intensity distribution was similar to recent results from the U.S. Army BMT, with the majority of activity classified as ‘sedentary’ or ‘light’ [25]. Consistent with steps, accelerometer-derived energy expenditure was also higher than a recent study of U.S. Army recruits (~18 MJ vs. ~14 MJ per day) [26] but similar to energy expenditure in British Army recruits measured by doubly-labelled water [21]. It is acknowledged that direct comparison to other BMT programs is difficult due to programmatic and methodological differences [27], but these previous findings help to contextualize the current findings. Regardless, these findings clearly demonstrate that Australian Army recruits are exposed to a high volume of physical activity during BMT, albeit mostly low intensity. This volume of physical activity is likely to represent a large increase in habitual activity levels for many recruits based on trends in the general population [25,28].

Challenging training stimuli followed by adequate recovery periods can elicit improved physical performance and decrease injury prevalence when compared with conditions of inadequate recovery [6]. On average, recruits’ physical fitness significantly increased from week 2 to week 8 of BMT, which suggests that the training stimuli imposed was sufficient to elicit positive adaptations for most recruits. This provides at least partial support for the effectiveness of the current BMT program to provide a periodized training stimulus with adequate recovery opportunity. However, there is some divergence in the prescribed weekly PT demand and the total physical demand for that week, particularly in weeks two to five. This observation is supported by previous studies showing that PT represents a relatively small proportion of the scheduled training program, and it is activities outside of PT (e.g., load carriage, field and combat training, drill, walking) that can impose greater training stress [2,9,29]. It has been noted that recruits appear to be most susceptible to injury during the first five to six weeks of BMT [30,31,32]; for example Hall [30] reported that 71% of injuries in British Army recruits occur within the first six weeks of BMT.

The current study observed a change in perception of effort over BMT, which is consistent with previous studies in recruits undertaking BMT [21] and conscripts undertaking field training [22]. However, rather than identifying weeks of intensified training demands, the current results revealed weeks of decreased perception of effort, appearing to offer recruits an opportunity to recover from the demands of BMT. High training demands have been shown to increase mood disturbance during military training [11,33], and symptoms of overreaching have been reported previously in recruit cohorts [11,12]. Consistent with these previous findings, the MTDS was sensitive to weekly variations in training demands. This was true for the total MTDS score and the individual subscales of depressed moods, vigor, physical signs and symptoms, and sleep disturbances. The progressive improvement in subjective wellness measures over the first four weeks is likely to represent a period of adjustment to the new training environment [34], and the transition from civilian life to the military training environment, which superimposes a variety of additional stressors on top of the physical demands [3,35].

MTDS total scores were lowest in weeks 6 and 12, which coincided with the weeks of lowest physical demands. This is similar to the findings of O’Leary et al. [21] who observed a moderate association between perceived training demands and distance travelled. In the same study, daily RPE was also associated with TRIMP and %HRR. In the current study, the greatest training distress (i.e., MTDS total score) was reported in week 10, which included the first half of the field training phase. Anecdotally, the field-training phase is the most challenging component of BMT for recruits as it involves disrupted sleep and nutrition combined with various physical and cognitive stressors. The results of the current study indicate that the MTDS total score reflects the strain associated with the multi-stressor training environment (i.e., PT, military skills and education classes, incidental PA, psychological and cognitive demands), not just the physical demands of training. Therefore, the results support the effectiveness of the multi-dimensional wellbeing questionnaire in capturing various factors that influence total subjective wellbeing.

The current findings support the potential utility of subjective wellbeing as a cost-and time-effective tool for the ongoing monitoring of recruits. It builds on the previous research of Ojanen et al. [12] who reported an increase in perceptions of exertion during multi-stressor military training. The observed increases in depressed moods with increases in training load, are consistent with previous literature examining the effects of high training demands, or potential overtraining, where the accumulation of stressors have the potential to exceed an individual’s capacity to cope [36,37]. Similarly, the relationship between self-reported physical signs and symptoms of training stress and indices of training demands in the current study are consistent with previous findings in military contexts. Increases in muscle soreness have been shown to increase when load and EE are increased during strenuous winter military training [38]. The lack of significance of light physical activity potentially supports the sensitivity of the MTDS physical signs and symptoms subscale.

## 5. Conclusions

In summary, the training demands imposed across Australian Army BMT varied across the 12 weeks and reflected intended periodization of training and recovery. The sixth and twelfth weeks provided an opportunity to recover from the multi-stressor training environment, as demonstrated by the lower physical demands combined with improved subjective wellbeing. Physical fitness improved from week 2 to week 8, indicating a positive training effect from the BMT program. Self-reported measures were sensitive to fluctuations in training stressors consistent with theoretical expectations. The MTDS exhibited significant associations with indices of training demands (i.e., light PA, moderate PA, weekly EE and RPE). Similar to applied sport contexts, subjective measures of stress responses may offer more utility for individual management of personnel than quantification of training stressors in the military training environment. The daily use of accelerometers and multi-domain self-report tools may, therefore, help to better manage how recruits are coping with the demands of training during BMT, providing opportunities for the early identification of recruits at risk of injury, maladaptation or attrition.

## Figures and Tables

**Table 1:** Descriptive data of participants.

Participant Variable	Value
N	48
Age	24.4 ± 7.1
Height (cm)	176.5 ± 9.9
Body Mass (kg)	76.8 ± 14.7

**Table 2:** Model of best fit for predicting MTDS total score.

Predictor Variable	Fixed Effect Measures		
	F	ß	*p*
Intercept	0.12	−2.093	0.734
NASA TLX	15.27	0.229	<0.001 *
Daily steps	9.70	0.001	0.002 *
% Vigorous activity	8.05	−140.175	0.005 *
% Moderate activity	3.37	−56.335	0.067
% Light activity	0.42	5.843	0.516
RPE	1.22	0.594	0.270
Gender	1.78	5.450	0.192
Push up performance change	1.53	−0.180	0.225
Model AIC	2157.519		

Note: NASA TLX, National Aeronautical Space Agency task load index; RPE, rating of perceived exertion; AIC, Akaike’s Information Criterion. * indicates significance at *p* < 0.01.

## Data Availability

The data are not publicly available. It may be possible to request access from the Defence Science and Technology Group, with appropriate ethical approval from the Department of Defence and Veterans’ Affairs Human Research Ethics Committee.
